# Lumbar Spinal Fusion Using Lateral Oblique (Pre-psoas) Approach (Review)

**DOI:** 10.17691/stm2021.13.5.09

**Published:** 2021-10-29

**Authors:** A.Ya. Aleinik, S.G. Mlyavykh, S. Qureshi

**Affiliations:** Neurosurgeon, Institute of Traumatology and Orthopedics Privolzhsky Research Medical University, 10/1 Minin and Pozharsky Square, Nizhny Novgorod, 603005, Russia; Director of the Institute of Traumatology and Orthopedics Privolzhsky Research Medical University, 10/1 Minin and Pozharsky Square, Nizhny Novgorod, 603005, Russia; Associate Attending Orthopedic Surgeon Hospital for Special Surgery, 535 East 70^th^ St., New York, NY, 10021, USA;; Associate Professor of Orthopedic Surgery Weill Cornell Medical College, 1300 York Avenue, New York, NY, 10065, USA

**Keywords:** lumbar fusion, anterior lumbar fusion, anterolateral lumbar fusion, retroperitoneal access, oblique lumbar interbody fusion, OLIF, anterior to psoas lumbar interbody fusion, ATP

## Abstract

Lumbar spinal fusion is one of the most common operations in spinal surgery. For its implementation, anterolateral (pre-psoas) approach (oblique lumbar interbody fusion, OLIF) is now increasingly used due to its high efficacy and safety. However, there is still little information on the clinical and radiological results of using this technique.

**The aim of the study** was to analyze the safety and efficacy of OLIF in the treatment of lumbar spine disorders as presented in the literature.

**Materials and Methods:**

The systematic electronic search was performed using the Ovid Medline, PubMed, and eLIBRARY.RU electronic databases. The following search key words were used: Oblique Lumbar Interbody Fusion, OLIF, Anterior to Psoas Lumbar Interbody Fusion, and ATP.

**Results:**

For the final analysis, 17 sources were selected; with a total of 2900 patients. Total complication rate was 13.9% (403 cases). The incidence of severe persistent complications was less than 1%. Based on the data obtained, we compared the clinical and radiological results of OLIF with other lumbar fusion methods.

**Conclusion:**

OLIF is an effective, versatile, and minimally traumatic option for lumbar fusion with relatively few complications, which makes it superior to other retroperitoneal approaches. However, the OLIF technique is not completely free of complications associated with the ventral approach, and it cannot provide adequate decompression of the spinal canal in all cases. In addition, anterior approach surgery is still of limited use in cases of spinal deformities; adequate correction of deformity is achievable mainly in combination with posterior surgery.

## Introduction

Lumbar interbody fusion is still the gold standard for stabilizing surgery in a wide range of lumbar spine disorders. However, there is no generally accepted technique of this operation; therefore, when choosing the optimal method, the surgeon has to consider their own technical skills and the patient’s needs.

Since the middle of the XX century, anterior lumbar interbody fusion (ALIF) has become a commonly used approach. This technique had a number of advantages including good restoration of the intervertebral space height and improvement of segmental lordosis with the formation of an adequate bone block. Nevertheless, the   implementation of this technique is associated with the risks of damage to large vessels, ureters, or abdominal organs; accordingly, the operation necessitates the involvement of abdominal and/or vascular surgeons. A number of authors recommend inviting general or vascular surgeons to perform the surgical access; this requirement has become mandatory in some countries [1]. With the current trend of highly specialized surgical intervention, this access is, therefore, becoming less popular among spinal surgeons.

The difficulties associated with ventral access to the spine can be avoided by using the traditional minimally invasive posterior approach commonly used by neuro-and orthopedic surgeons. The first description of the posterior lumbar interbody fusion (PLIF) was presented by Briggs and Milligan in 1944 [2]. Later, transforaminal lumbar interbody fusion (TLIF) was developed to become the most common procedure by the end of the last century. This operation, introduced by Harms and Rolinger [3], makes it possible to perform interbody fusion using the regular posterior access, while performing “direct” decompression of the spinal canal without the need for significant traction of the dural sac and spinal roots. Thanks to the ease of access and other technical options, this type of lumbar fusion remains the most commonly used by now.

However, direct access to the spinal canal is associated with a risk of damage to the dural sac, spinal roots, or epidural vessels; in addition, with the posterior approach, mechanical trauma to the paravertebral muscles is more likely. Therefore, when possible, most surgeons practice minimally invasive surgery (miniTLIF) to reduce the risk of surgical trauma.

Another disadvantage of posterior fusion options is the gradual kyphosis of the operated segment caused by the cage subsidence into vertebral bodies; moreover, the risk of developing pseudoarthrosis in PLIF is slightly higher than that with ALIF [4].

Recently, direct lateral interbody fusion (DLIF) has been developed and introduced into surgical practice. The terms XLIF, ELIF (extreme lateral interbody fusion) are used to denote the direct lateral access. In this case, minimally invasive access to the lateral surface of the intervertebral disc is provided through the psoas muscle. This approach allows one to perform interbody fusion (with minimal surgical trauma) and correct frontal spinal deformities using large support cages. However, evidence has accumulated that using this access, even under neurophysiological monitoring, is associated with a high risk of damage to the lumbar plexus. The resulting neurological deficit was observed in as many as 75% of operated patients [5–9].

To reduce the likelihood of these complications, the lateral approach was modified into the indirect retroperitoneal access or pre-psoas approach (oblique lumbar interbody fusion, OLIF or anterior to psoas lumbar interbody fusion, ATP). In this technique, access to the disc is obtained through the anatomical window between the large vessels and the psoas muscle. The method was first described by Mayer in 1997 [10], but the term OLIF was introduced much later — in 2012 in the work of Silvestre et al. [11]. The anatomical features of the access trajectory were studied in detail, and safe operation at L_1_ to S_1_ levels was demonstrated [12]. These data were confirmed by MRI studies [13]. The surgical aspects of the retroperitoneal space were also described in detail [14].

Thus, OLIF is currently one of the optimal options for lumbar fusion, combining high efficiency and safety for the patient. However, to date, there are few studies evaluating the radiological and clinical outcomes of this technique. In the Russian-language literature, there are no such publications at all.

**The aim of the study** was to analyze the safety and efficacy of OLIF in the treatment of lumbar spine disorders as presented in the literature.

## Materials and Methods

The search was performed in the Ovid Medline, PubMed, and eLIBRARY.RU electronic databases using the key words: Oblique Lumbar Interbody Fusion, OLIF, Anterior to Psoas Lumbar Interbody Fusion, and ATP. For inclusion in the full-text analysis, the publications were selected according to the following criteria: 1) the study should include patients who underwent lumbar interbody fusion using the OLIF technique at one or more levels in combination with minimally invasive fixation; 2) the study endpoints should include imaging and/or clinical data reflecting one or more below indicators:

the incidence of complications and their structure;

the duration of the operation, surgical blood loss, the length of hospital stay;

the clinical results: the level of pain, the level of disability by the Oswestry questionnaire;

the radiological data: recovery of the disc height, segmental lordosis, improvement of the sagittal balance, occurrence of successful formation of the bone block, as well as an increase in the cross-sectional area of the spinal canal.

Our full-text analysis did not include studies with small numbers of patients and studies where fundamentally different surgical techniques were used. Only English-and Russian-language articles were screened.

**Figure 1.**
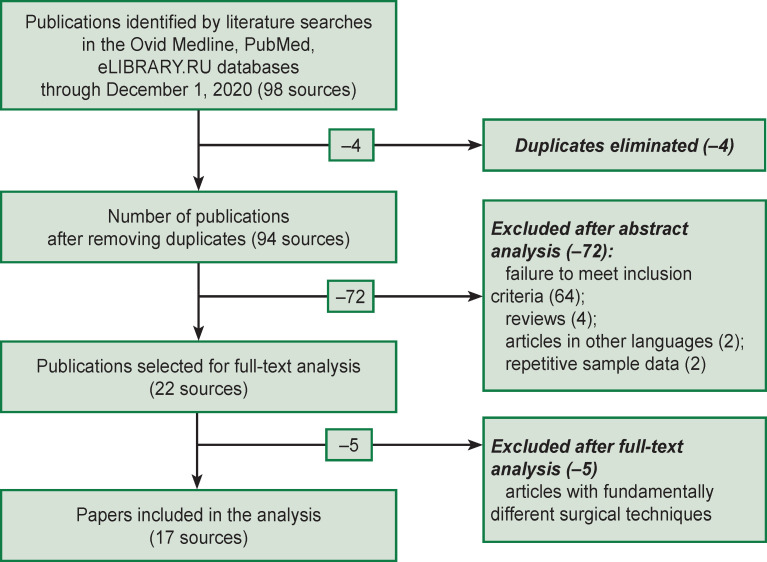
Flowchart of the literature search

The selection of articles and their analysis was carried out independently by two specialists who had over 10 years of experience in spinal surgery. The most recent search was conducted on December 1, 2020. At the first stage, the search based on the above key words resulted in 98 publications. At the second stage, the study titles were analyzed, duplicate titles excluded, and 94 sources were selected for further consideration. At the third stage, we analyzed the abstracts of the selected papers; here we excluded articles that did not meet the inclusion criteria, literature reviews, repeated publications of the same results, and articles in languages other than Russian or English (22 full-text papers were selected for the final analysis). At the final stage, articles, in which the surgical technique was fundamentally different from the classical one were eliminated. When the two co-authors disagreed on including/excluding a publication, a joint discussion with the involvement of additional experts was held. The flowchart of this search is shown in Figure 1. Articles selected for full-text analysis (17 sources) are characterized in Table 1.

**Table 1:** Articles included in the analysis

Study	Year	LE	Number of patients	Number of segments	Average age (years)	Diagnosis	Follow-up period (months)	Indicators	Complication rate (%)
Patel et al. [15]	2010	3	23	36	61	DDD, DDEF	24	SFC	21.70
Silvestre et al. [11]	2012	3	179	318	54.1	DDD, DH, SL, SS, DDEF	12	SFC	11.73
Ohtori et al. [16]	2015	3	35	51	67	SL, SS, DDEF	6	SFC, CR	20
Fujibayashi et al. [17]	2015	3	28	52	65.3	DDD, SS	After surgery	SFC, RR	28.50
Mehren et al. [18]	2016	4	812		63	DDD	After surgery	SFC	3.70
Abe et al. [19]	2016	3	155		63.5	DDD	After surgery	SFC	48.30
Molloy et al. [20]	2016	3	64	120	63.5	DDD	18	SFC, CR	40.60
Gragnaniello, Seex [21]	2016	3	24	32	62.4	DDD, SL	6	SFC, CR	33.0
Woods et al. [22]	2017	4	137	340	62	DDD, SL, SS, DDEF	6	SFC, RR	11.70
Sato et al. [23]	2017	3	20	20	69	SL	6	SFC, RR	25.0
Lin et al. [24]	2018	3	25	25	64	SS, SL, DDD at L_4_–L_5_ level	24	SFC, CR, RR	36.0
Zeng et al. [25]	2018	3	144		61.9	DDD, DH, SL, SS, DDEF	15	SFC, CR, RR	32.34
Miscusi et al. [26]	2018	3	14	18	57.4	DDD	38	SFC, CR, RR	7.10
Jin et al. [27]	2018	3	63	93	67.1	DDD	18	SFC, CR, RR	28.57
Tannoury et al. [28]	2019	4	940	2429	58.9	DDD, SL, SS, DDEF	12	SFC	8.20
Chang et al. [29]	2019	3	169	262	67.7	DDD, SL, SS, DDEF	12	SFC, CR, RR	16.60
Beng et al. [30]	2019	3	28	28	74	DDEF	After surgery	SFC, RR	17.86

Here: LE — level of evidence; DDD — degenerative disc disease; SL — spondylolisthesis; DDEF — degenerative deformity; SS — spinal stenosis; DH — disc herniation; SFC — structure and frequency of complications; CR — clinical result; RR — radiological result.

## Results and Discussion

### Structure and frequency of post-surgery complications

Table 2 shows the structure and frequency of complications associated with surgical intervention as presented in the selected publications.

**Table 2:** The frequency and structure of OLIF-associated complications

Study	Year	Number of patients	Intervention at level L_5_–S_1_	Average age (years)	Follow-up period (months)	Total number of complications (%)	Complications (%)													
							Infectious	Vascular	Retrograde ejaculation	Intestinal paresis	Ureteral injury	Motor	Sensory	Hip pain	Sympathectomy	Dm injury	Pseudoarthrosis and screw instability	Vertebral fracture	Cage subsidence	Reoperations
Patel et al. [15]	2010	23	0	61	24	21.70											4.35 (1 case)			0
Silvestre et al. [11]	2012	179	1	54.1	12	11.73		1.68		0.56			1.12	2.20	1.70		0.56			0.56
Ohtori et al. [16]	2015	35	0	67	6	20		2.86				2.86	8.57	2.86					2.86	
Fujibayashi et al. [17]	2015	28	1	65.3	After surgery	28.50							7.14	7.14						
Mehren et al. [18]	2016	812	0	63	After surgery	3.70	0.62	0.37		0.24		0.12	0.24	0.24						
Abe et al. [19]	2016	155	1	63.5	After surgery	48.30	1.90	2.60			0.65	1.2	13.5						18.70	1.90
Molloy et al. [20]	2016	64	1	63.5	18	40.60	3.10			12.50		4.70				6.30				4.70
Gragnaniello, Seex [21]	2016	24	0	62.4	6	33	4.17					12.50	8.30							4.17
Woods et al. [22]	2017	137	1	62	6	11.70			0.70	2.90									4.40	
Sato et al. [23]	2017	20	0	69	6	25		5					5	5					10	
Lin et al. [24]	2018	25	0	64	24	36		4					8	12	8					
Zeng et al. [25]	2018	235	0	61.9	15	32.34		2.98		0.85		5.11	2.98	2.98	1.28			1.28	9.36	
Miscusi et al. [26]	2018	14	0	57.4	38	7.10						5							0	
Jin et al. [27]	2018	63	0	67.1	18	28.57					1.59		14.29	3.18	6.35	1.59				
Tannoury et al. [28]	2019	940	1	58.9	12	8.20	0.20	0.30	0.20	1		0.95	2.60	0.50		0.50		0.30		1.50
Chang et al. [29]	2019	169	1	67.7	12	16.60	0.60	1.80				0.60					1.20		32	
Beng et al. [30]	2019	28	0	74.12	After surgery															

Here: DM — dura mater.

We noted wide variations in the total number of observed complications — from 3.7% [22] to 48.3% [19] (see Table 2); among those, the frequency of persistent complications was 1.9%. Considering all patients included in the review (n=2900), 403 complications (13.9%) were identified. For comparison, the average number of all complications according to published meta-analyses was 19.25% for TLIF, 31.4% for ELIF [31], and 14.1% for ALIF [32].

Although the development of OLIF was aimed at avoiding the complications typical for ALIF and ELIF, some of these events occur also with the OLIF technique. For instance, most authors note the possibility of vascular complications (frequency — from 0 to 5%), although in many cases, the damage occurs to segmental vessels and does not entail serious consequences; the number of cases with damage to large vessels is less than 1% [23, 24].

At the same time, in ALIF, damage to the large vessels occurs in 2.9–7.2% of cases, which significantly increases the risk of fatal consequences; therefore, the participation of a vascular surgeon is often needed to access the intervertebral disc [33, 34]. With OLIF, access is usually performed by a spinal surgeon.

After OLIF, retrograde ejaculation could be observed in some cases in male patients (0.2–0.7%). This complication is considered typical for ALIF, with the frequency exceeding 1.7%, especially for the L_5_–S_1_ level operations [35]. In OLIF, the risk of this complication is minimal and has been described only in two studies with large patient populations [22, 28]. However, despite the low incidence, the likelihood of this complication should be taken into account when planning the OLIF operation at the L_5_–S_1_ level, especially in young men.

In addition, damage to such retroperitoneal organs as the ureters was described (the frequency did not exceed 1.59% and was not identified in most studies). Specifically, a clinical case of secondary ureteral stenosis with the development of kidney atrophy has been published. Nevertheless, the risk of this complication should also be taken into account and renal function should be monitored for a long time, especially in the presence of radiographic signs of cage displacement beyond the vertebral body boundaries [36].

A frequent early postoperative complication after retroperitoneal access is intestinal paresis, which represents a normal physiological response to surgery within 48 h, and after this period is considered pathological. With OLIF, the incidence of pathological intestinal paresis can reach 12.5%; however, in some patients, this complication has not been reported at all. Apparently, not all authors interpret temporary intestinal paresis as a complication [37].

An extremely heavy complication for the patient and the surgeon after spinal fusion is the development of a new neurological deficit. According to the literature [5–9], most often this complication occurs when the access is made through m. psoas (ELIF); the complication is associated with injury to the lumbar plexus, which runs along the surface and in the thickness of the psoas muscle. In this case, the risk of motor deficit can reach 20–36%, sensory impairments — 25–75%, and pain in the anterior thighs — 23–60%. The majority of proponents of this approach recommend using neurophysiological monitoring during surgery, but even so, the frequency of neurological complications remains relatively high — 8.1% [38].

With OLIF, the risk of damage to the lumbar plexus also exists, but it is significantly lower, since access is made through the anatomical corridor bypassing the lumbar muscle and nerve trunks of the lumbar plexus.

The risk of developing a temporary motor deficit is 0–12.5%, sensory impairments — 0–14.3%, pain in the anterior surface of the thigh — 0–12% [21, 24, 27, 39]. In addition, with this approach, there is a risk of a specific neurological complication — damage to the sympathetic nerve cord, clinically manifested in an increase in the temperature of the lower limb on the ipsilateral side of the access. The incidence of this complication can be as high as 8%. However, in most cases, such manifestations are temporary and disappear within a few weeks; with the access at three or more levels, symptoms may persist longer [24].

In posterior approaches (PLIF, TLIF), the structure of neurological complications is significantly different, primarily due to the impact on the spinal roots in the area of the intervertebral foramen and on the dural sac in the spinal canal. The incidence of temporary neurological deficits can reach 20.16%, persistent sensitive deficits — 2.22%, and persistent motor deficits — 1.01% [31]. These complications are mainly caused by damage to the dura mater and improper positioning of the screws [40]. According to some authors, with OLIF, damage to the dura mater is observed in 6.3% of cases [20]. This is likely due to the technical specifics of the operation, since most authors do not note such complications. In the classical version, this approach does involve an access to the spinal canal, and the required decompression is achieved by an indirect method. Along with that, the technique of direct ventral microsurgical decompression of the spinal canal during OLIF was described [41]. With this technique, there is a risk of injury to the dura mater, but the option of direct decompression gives the OLIF an advantage over ELIF, when only indirect decompression is possible.

A significant problem with the interbody fusion intervention is the cage subsidence into the vertebral bodies due to damage to the endplates. Thus, according to a meta-analysis [42], the incidence of this complication in lateral fusion is 10%, and in 2.7% of cases, it requires reoperation. The OLIF technique is not without a risk of this complication: its frequency can reach 32% [29]. In most studies though, it does not exceed an average of 10% [22, 25, 26]; considering all the cases reported, we assume it is 3.9%. This value is similar to that obtained for ELIF. Apparently, the high incidence of damage to the endplates in some studies is due to the fact that the authors used CT scans to verify subsidence, which revealed small degrees of subsidence not detectable with X-ray. In TLIF, the subsidence rate is significantly higher — 15.9% [43], which is associated with a smaller area of the cage support [44]. According to some authors, with lateral fusion, there is a significant risk of damage to the endplates when the surgeon installs a large cage in patients with reduced bone density. Intraoperative damage to the endplates was noted in more than 10% of cases [45]. The lowest risk of this complication was observed with ALIF, since with the ventral approach, a wide release of the anterior longitudinal ligament is performed, which ensures maximum accessibility and mobility of the intervertebral space; the cages installed with this access have the largest support area. Even in the absence of dorsal fixation, cage subsidence of more than 2 mm was observed in 10.2% of patients only [46].

The risk of developing pseudoarthrosis is one of the most important characteristics of the technique for spinal fusion. With OLIF, the incidence of pseudarthrosis does not exceed 1.2% [29]. The formation of complete bone fusion is noted in 94.9% of patients as early as 6 months after surgery [22]. Notably, our analysis includes results obtained with OLIF in combination with dorsal screw fixation. Along with that, OLIF can be performed with ventral fixation [47] or with no fixation [48]; in those, although the risk of developing pseudarthrosis and subsidence increases, the invasiveness decreases, and complications associated with screw fixation are ruled out.

In ALIF with screw fixation, spinal fusion is also successfully formed in most patients — up to 97.4% [49]. With TLIF, the success rate of bone block formation is also high (according to meta-analysis [40]), but somewhat inferior to the ventral approaches: with TLIF — 94.8%, with miniTLIF — 90.9%, which can be explained by the smaller contact area of the vertebrae with the bone grafts.

The level of infectious complications in OLIF is minimal and does not exceed 1% in large patient populations [14, 18, 50]. For comparison, in the case of TLIF, the incidence of infectious complications is significantly higher: from 12% with mini-access and up to 25% with open access [41].

The frequency of revisions after OLIF in large groups of patients is extremely low: 0–1.9% [18, 28, 50], in small groups, it can be 4–5%.

An important factor is that the OLIF method is relatively new. Among the analyzed publications, we found no studies with a follow-up period of longer than three years, and most of them were limited to a period of 12 months. For this reason, the structure of complications does not include late complications described for other types of fusion, such as adjacent segment disease or fixator instability.

The analysis shows an inverse relationship between the total number of complications and the number of patients included in the study. This fact is most likely associated with the “learning curve”: in those clinics where spinal fusion surgeries are performed more often and surgeons have more experience, complications are significantly less frequent. The study by Liu and Wang [51] showed that the operation time and the number of complications significantly decreased after the first 25 operations.

### Assessment of surgical trauma

The most common indicators of surgical trauma are surgical blood loss, duration of surgery, and length of hospital stay. In the analyzed articles, these indicators are mentioned in 7 reports (Table 3).

**Table 3:** OLIF-associated trauma

Study	Year	Number of patients	Number of segments	Intervention at level L_5_–S_1_	Average age (years)	Follow-up period (months)	Operation time (min)	Blood loss (ml)	Hospital stay (days)
Patel et al. [15]	2010	23	36	0	61	24		Less than 200	4.1
Silvestre et al. [11]	2012	179	318	1	54.1	12	32.5	57	7.1
Fujibayashi et al. [17]	2015	28	52	1	65.3	After surgery	72.5	17	
Molloy et al. [20]	2016	64	120	1	63.5	18	62	33	
Woods et al. [22]	2017	137	340	1	62	6		82	
Lin et al. [24]	2018	25	25	0	64	24	95.96	106.4	8.52
Jin et al. [27]	2018	63	93	0	67.1	18	122	253	6.8

With OLIF, the average operation time varied from 32.5 to 122.0 min (in studies with minimal indicators, these numbers pertained to the time spent for operating on one segment and not the total time of the operation). Blood loss was 17–272 ml (when fusion was performed on one segment). The duration of hospital stay ranged from 4.1 to 8.5 days.

With miniTLIF, the operation time was 116–390 min, with TLIF — 102–365 min; blood loss — 51–578 and 225–961 ml, respectively; hospital stay — 2.3–10.6 and 2.9–14.6 days, respectively [40]. With ALIF, the average blood loss is 122 ml, the operation time is 89 min, and the hospital stay is 5.3 days [52]. Thus, in the studies analyzed, the surgical trauma with OLIF is less pronounced than that with TLIF and ALIF, as evidenced by comparative studies of these techniques [24, 53–55]. With ELIF, the results do not differ significantly from the data on OLIF.

### Assessment of clinical results

Until now, a generally accepted criterion for assessing the clinical outcome in spinal surgery has not been developed. However, the most commonly used tools are the visual analogue scale (VAS) and the Oswestry Disability Index. Among the selected studies, the clinical efficiency of operations was assessed in 9 studies, all of them showed significant improvements in the scores related to these scales [16, 20, 21, 23, 24, 26, 27, 29, 56].

**Figure 2.**
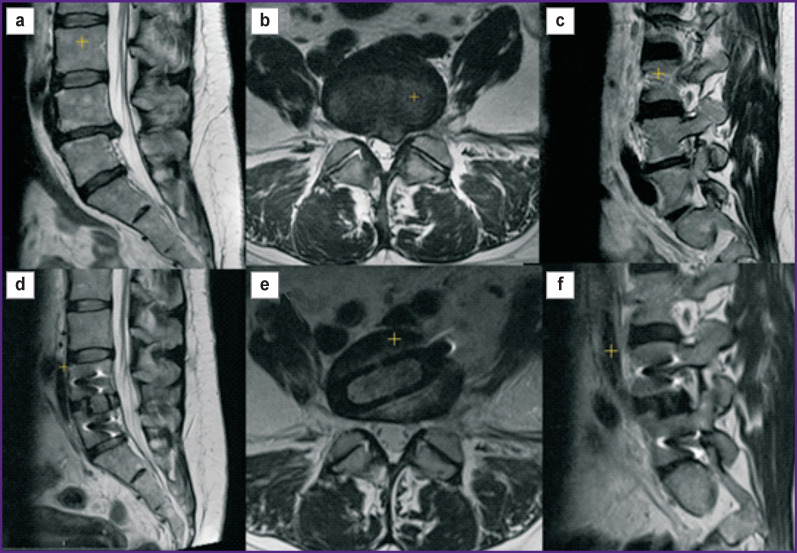
Indirect decompression in OLIF Female patient 52 years old, with recurrent disc herniation at L_4_–L_5_, instability of the L_4_–L_5_ segment, radiculopathy of the L_5_ vertebra on the left, and chronic vertebral pain syndrome. MRI image shows the sagittal section in the central part of the spinal canal: (a) before surgery, (d) after OLIF; axial section through the L_4_–L_5_ disc: (b) before surgery, (e) after OLIF; sagittal section at the level of the left intervertebral foramen: (c) before surgery, (f) after OLIF

Adequate decompression of the spinal canal during stenosis is a major factor contributing to the clinical effect; only decompression can lead to regression of persistent neurological symptoms. With OLIF, decompression is achieved due to the so-called effect of indirect decompression, i.e. an increase in the cross-sectional canal area due to the restoration of the anatomical relationships in the spinal motion segment. Figure 2 shows a clinical example of using OLIF for achieving decompression of the spinal canal and intervertebral foramen.

As previously shown for ELIF, the degree of canal area enlargement correlates with the clinical effect in patients with lumbar stenosis [57]. This aspect is addressed in three of the studies included in the review [17, 23, 30] (Table 4). According to their data, the change in the cross-sectional area of the spinal canal increases from 19.0 to 60.4%; these numbers correlate with the increase in the height of the intervertebral disc, which averages at 4.5 mm, or 45–61% of the initial height. There is also a proportional increase in the size of the intervertebral foramen. The most pronounced effect is observed in patients with preserved lumbar lordosis (before surgery); when lordosis is lost or lumbar kyphosis is present, indirect decompression is less effective [23]. In a review on ELIF [55], it was shown that the most significant effect of indirect decompression was observed in foraminal stenosis (an increase in the area of intervertebral foramina up to 35%), while in central and subarticular stenosis, the results were less impressive and not confirmed in all studies. For this reason, in such cases, it is recommended to use rear decompression to supplement the OLIF.

**Table 4:** Radiological changes after OLIF

Study	Year	Number of patients	Number of segments	Intervention at level L_5_–S_1_	Average age (years)	Increase in spinal canal area	Angle correction (degrees)	Increase in disc height	Sagittal balance
Fujibayashi et al. [17]	2015	28	52	1	65.3	30.20%	4.5	4.5 mm	
Molloy et al. [20]	2016	64	120	1	63.5				ΔPT — 7°, ΔSS — 8°, ΔLL — 19°, ΔSVA — 5 cm
Sato et al. [23]	2017	20	20	0	69	19%		61%	
Miscusi et al. [26]	2018	14	18	0	57.4		2.5		
Jin et al. [27]	2018	63	93	0	67.1		5	4.5 mm	
Beng et al. [30]	2019	28	28	0	94	27.5% (LL<0°), 32.1% (0°<LL<20°), 60.4% (LL>20°)		45.30%	

Here: PT — pelvic tilt; SS — sacral slope; LL — lumbar lordosis; SVA — sagittal vertical line drawn through C_7_.

### Evaluation of radiological results

It has been established by now that a significant factor influencing the results of spinal fusion is the restoration of geometric proportions in the spinal motion segments (see Table 4). Only a few studies included in this review provide data on angular correction in OLIF [17, 26, 27]; according to these reports, the angular correction is 2.5–5.0° per segment. This aspect of spinal fusion was studied in more detail for ELIF, for example, in the work of Park et al. [58]. They showed the effect of cage position on the angular correction and the spinal canal area. Thus, when the interbody implant is located in the anterior third of the disc, the highest angular correction (>6°) is reached without negatively interfering with indirect decompression.

Only one study included in the analysis showed the effect of OLIF on the parameters of global sagittal balance and spinal-pelvic relationship [20]. Correction of the main parameters, according to this study, is: ΔPT — 7°, ΔSS — 8°, ΔLL — 19°, and ΔSVA — 5 cm.

In the analyzed studies, no correlation was observed between the OLIF procedure and frontal deformity. Here, we do not discuss studies narrowly focused on evaluating OLIF as a tool for correcting spinal deformity. For example, the work of Park et al. [59] assesses the possibilities of multilevel OLIF in correcting the sagittal balance without using posterior osteotomy. However, the authors emphasize that in minimally invasive transpedicular fixation, they applied special methods to correct the deformity, such as configuring the operating table to enhance lumbar lordosis, bending the rods, and using the extensive screw-to-screw compression.

In most studies on the role of OLIF in the correction of spinal deformities, the authors emphasize the need for posterior osteotomies to achieve an adequate degree of correction. Thus, Kim et al. [60] found that although interbody fusion had some impact on balance correction, the main corrective effect could be achieved only after performing a dorsal approach using osteotomy of the articular processes or even a three-column osteotomy combined with transpedicular fixation. For the correction of rigid deformities, a three-stage treatment with OLIF has also been proposed: at the first stage, posterior release (osteotomy of the articular processes) is performed, at the second stage, multilevel OLIF, and at the third stage, final correction and transpedicular fixation [61]. Only such a multi-stage approach can provide adequate correction of rigid deformities through anterior fusion.

In conclusion, we would like to note the versatility of this method of fusion: it can be used for a wide range of degenerative diseases of the lumbar spine, deformities, traumatic injuries, infections, and tumor lesions [62, 63].

## Conclusion

OLIF is an effective, versatile, and minimally traumatic procedure for lumbar fusion with a relatively small number of complications, which makes this approach superior to the previously described TLIF, ELIF, and ALIF techniques. However, OLIF is not completely free of difficulties associated with the retroperitoneal access (like ALIF and ELIF), which should be taken into account when planning the operation. In addition, it is not always possible to provide adequate decompression of neural elements in lumbar stenosis; then, additional posterior decompression is required. The issue of using OLIF as the main tool for correcting spinal deformities remains unresolved. The corrective capabilities of ventral surgery are limited; instruments for anterior fixation are yet to be developed to become comparable to transpedicular instrumentation. By now, dorsal or dorsal-ventral accesses play the leading role in spinal fusion surgery.
